# Vascularized Iris Mass as Sentinel Manifestation of Syphilis in Patient with HIV Infection, Spain, 2025

**DOI:** 10.3201/eid3207.260388

**Published:** 2026-07

**Authors:** Marta Caminal-Caramés, Jaume Sánchez-Serra, Jesus Díaz-Cascajosa, Albert Saladrigas, Santiago Conversa, Jose Ignacio Vela-Segarra

**Affiliations:** Hospital Sant Pau, Barcelona, Spain

**Keywords:** Treponema pallidum, syphilis, sexually transmitted infections, bacteria, viruses, HIV/AIDS and other retroviruses, iris mass, iris papulosa, ocular syphilis, ultrasound biomicroscopy, optical coherence tomography, Spain

## Abstract

Vascularized iris masses are rare, yet highly suggestive of syphilis. We report a 51-year-old man in Spain with HIV infection who had painful vision loss, rash, and an iris mass. Laboratory testing confirmed syphilis; ocular manifestations resolved with intravenous ceftriaxone and penicillin. Early recognition of syphilis can prevent vision loss and neurologic complications.

Syphilis has experienced an alarming global resurgence over the past 2 decades, evolving into a major public health concern across developed nations (*1*). Ocular syphilis, known as the great masquerader, can involve any ocular structure and can occur at any stage of the disease; ocular syphilis frequently serves as the sentinel manifestation of an undiagnosed HIV infection (*2*,*3*). Although uveitis is the most common manifestation of ocular syphilis, vascularized iris masses, spanning a clinical spectrum from iris roseola and papulosa to iris nodosa and gummata, represent an exceptionally rare and poorly characterized phenotype (*4*). Fewer than 15 cases have been reported in English-language literature (*5*–*8*), and paradoxically, despite the synergy between syphilis and HIV infection, iris lesions are even more infrequent in persons with advanced immunosuppression (*9*). 

The formation of structured nodules or gummata typically represents an inadequate or failed delayed-type hypersensitivity (DTH) reaction (*8*). Differential diagnosis is challenging because the lesions can mimic other inflammatory, neoplastic, or opportunistic fungal processes in patients with HIV. Multimodal ultrasound biomicroscopy (UBM) imaging and anterior segment optical coherence tomography (AS-OCT) can aid in lesion characterization, defining depth, internal composition, vascularity, and stromal involvement (*5*,*8*). We report a case of iris papulosa as a sentinel sign of syphilis in a patient in Spain with HIV infection.

## The Case

**Figure 1:**
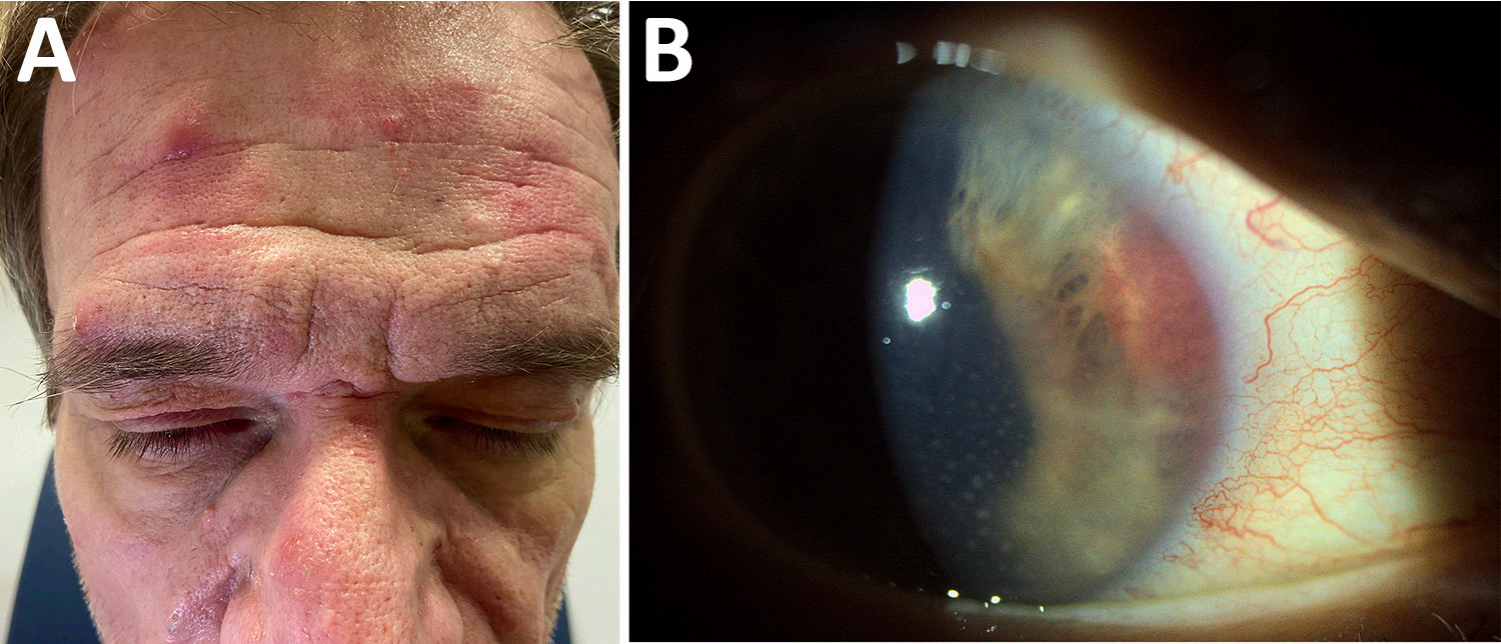
Clinical findings of a vascularized iris mass as sentinel manifestation of syphilis in patient with HIV infection, Spain, 2025. A) Maculopapular facial rash consistent with secondary syphilis. B) Slit-lamp photograph demonstrating a nonpigmented, vascularized iris base mass with conjunctival hyperemia, corneal edema, and mutton-fat keratic precipitates.

**Figure 2:**
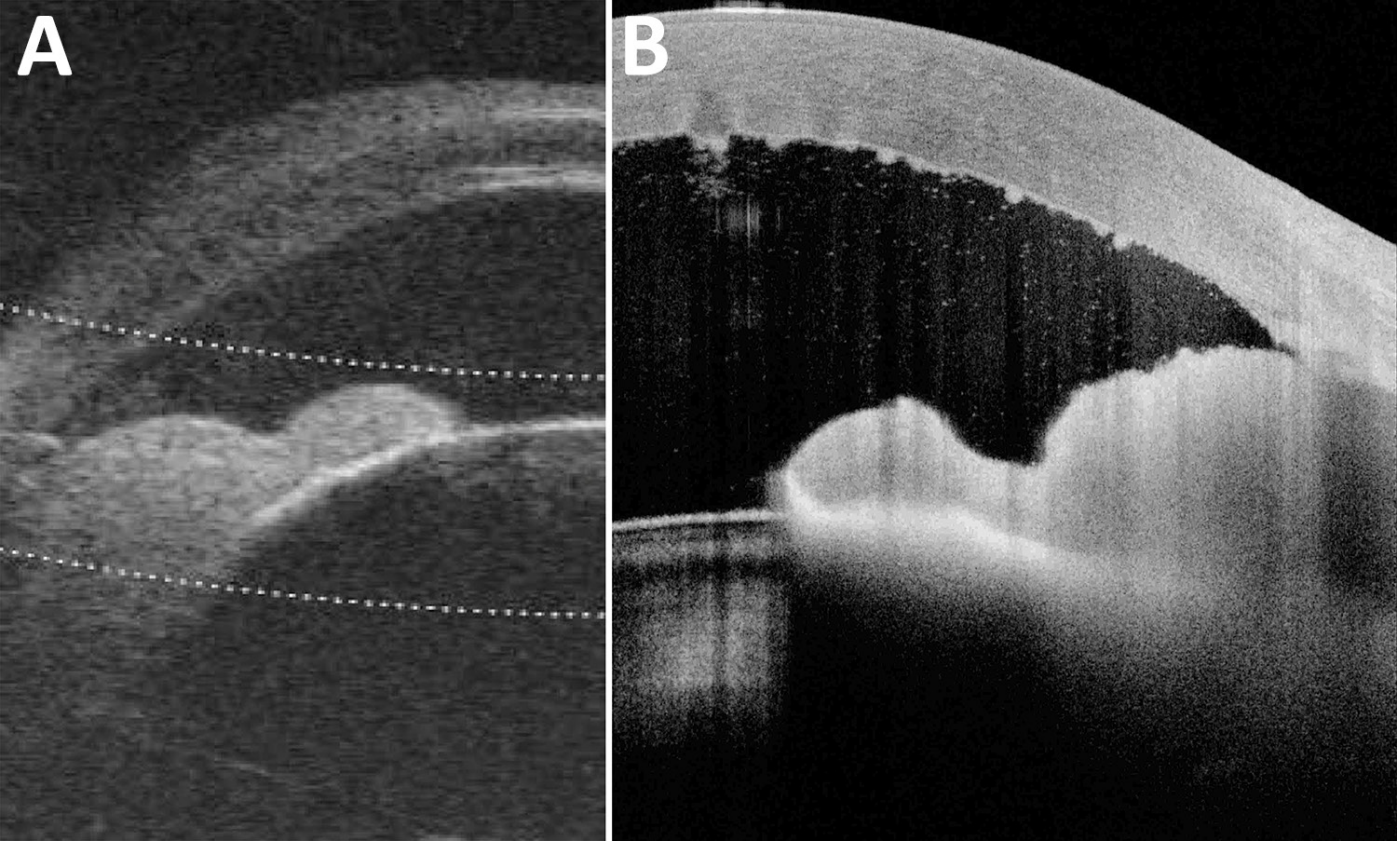
Imaging for a vascularized iris mass as sentinel manifestation of syphilis in patient with HIV infection, Spain, 2025. A) Ultrasound biomicroscopy revealing a well-defined hyperechoic iris mass with ciliary body involvement. B) Anterior segment optical coherence tomography showing a homogeneous hyperreflective stromal lesion with keratic precipitates and inflammatory cells in the anterior chamber.

In 2025, a 51-year-old man with a history of HIV, depression, and substance abuse (amphetamines and cocaine) sought care for 3 days of right-eye pain and vision loss. Facial maculopapular rash suggested secondary syphilis (Figure 1, panel A). Best-corrected visual acuity was hand motion in the right eye and 20/20 in the left eye. Intraocular pressures were 8 mm Hg in the right eye and 10 mm Hg in the left eye. Slit-lamp examination of the right eye revealed marked conjunctival hyperemia, a vascularized nonpigmented iris base mass, corneal edema, mutton-fat keratic precipitates, posterior synechiae, and 3+ anterior chamber cells and flare (Figure 1, panel B). Ocular ultrasonography ruled out vitreous inflammation and retinal detachment. UBM revealed a well-defined, homogeneous hyperechoic iris stromal mass with ciliary body involvement and iris pigment epithelium disruption (Figure 2, panel A). AS-OCT demonstrated a homogeneous lesion with irregular hyperreflective anterior surface, internal hyperreflectivity, posterior shadowing, iris pigment epithelium disruption, keratic precipitates, and anterior chamber cells (Figure 2, panel B).

Ocular syphilis was confirmed by rapid plasma regain (titer 1:256) and positive *Treponema pallidum* microparticle enzyme immunoassay. Lumbar puncture was performed, and cerebrospinal fluid tested *T. pallidum–*negative. HIV viral load was 25 copies/mL; CD4+ cell count was 13% (300 cells/μL). HIV history was unavailable because of poor follow-up; the patient reported intermittent, poorly adherent antiretroviral therapy but was taking it during uveitis diagnosis, which explains viral suppression despite persistent immunosuppression. No viral loads or CD4 counts were available from the previous year. Serologic tests for tuberculosis, sarcoidosis, hepatitis, and toxoplasmosis were negative. 

We administered intravenous ceftriaxone (2 g/d for 5 d) as an initial bridging therapy at home while we arranged logistics for in-home intravenous penicillin G (18 million units/d for 10 d) (*10*). Adjunctive therapy included 1% topical prednisolone acetate (4×/d), 1% cycloplegic agent (atropine; 2×/day, and systemic prednisone initiated at 30 mg/day and tapered over 2 weeks. At 1 month, best-corrected visual acuity recovered to 20/20, and repeat UBM and AS-OCT confirmed complete lesion resolution in the right eye.

Ocular syphilis most commonly occurs during the secondary stage of *T. pallidum* infection (*6*). Among its diverse phenotypes, vascularized iris masses, including iris roseola, papulosa, nodosa, and gummata, are considered exceptional but highly suggestive of treponemal disease (*5*,*6*). Such lesions are thought to result from *T. pallidum* capillary engorgement or focal inflammatory infiltration, representing a spectrum of progressively intensifying inflammatory responses (*6*). Iris roseola, the earliest manifestation in secondary syphilis, consists of dilated capillaries or vascular tufts and can evolve into iris papulosa, single or multiple nodules, and subsequent iris nodosa, reflecting increasing granulomatous stromal infiltration (*5*,*7*,*11*). In contrast, iris gummata typically occur in later stages, presenting as poorly vascularized nodules with central degeneration that represent chronic granulomatous inflammation associated with impaired cellular immune control (*5*,*6*,*8*).

In a comprehensive review of reports published in English, we identified only ≈13 cases of vascularized iris masses documented since 1915 (*5*–*9*) (Appendix Table). One case of a large syphilitic iris granuloma in a patient with HIV infection has been highlighted in recent reviews, whereas most published cases occurred in patients without HIV or with unreported HIV status (*9*) (Appendix Table).

The co-occurrence of syphilitic iris nodules and HIV infection appears paradoxically rare. Despite the epidemiologic synergy between HIV and syphilis, iris granulomas are reported less often among persons with HIV infection than in immunocompetent hosts (*12*). Gummata represent chronic granulomatous lesions arising from a failure of effective DTH reaction to *T. pallidum*, reflecting impaired cellular immune control (*8*). In HIV infection with CD4+ depletion, the immune system might lack the capacity for such granulomatous responses (*13*). Consequently, syphilis in patients with HIV more frequently manifests as severe, diffuse inflammation or posterior segment involvement, rather than discrete iris nodules (*12*,*13*). The pronounced granulomatous iris mass in this patient, despite CD4+ of 300 cells/μL, suggests residual capacity for granuloma formation despite DTH impairment, possibly due to partial immune reconstitution or localized immune responses.

In patients with HIV, a solitary vascularized iris mass substantially broadens the differential diagnosis. Syphilitic nodules must be distinguished from neoplastic entities, other infections, or inflammatory causes (*8*,*14*). Among neoplastic causes, iris nevus typically presents as a flat or minimally elevated, well-circumscribed pigmented lesion with slow growth and is usually asymptomatic, whereas iris melanoma tends to be more elevated and might exhibit intrinsic vascularity, corectopia, uveal ectropion, and progressive enlargement. Iris metastases are typically amelanotic and often identified in patients with a known primary tumor (*15*). Intraocular lymphoma, although less common, can appear as diffuse iris infiltration, often accompanied by chronic inflammation. Infectious causes of iris granulomas include ocular tuberculosis in the setting of chronic granulomatous uveitis, often with systemic involvement (*5*,*15*).

Anterior segment imaging can support diagnostic evaluation by helping define lesion depth and internal characteristics, thereby contributing to the differential diagnosis among inflammatory, neoplastic, and alternative infectious causes. However, the diagnosis of ocular syphilis remains primarily clinical and serologic, and imaging findings can further aid in lesion characterization and monitoring treatment response (*5*,*6*).

## Conclusions

Vascularized iris masses represent an exceptionally rare manifestation of ocular syphilis; only ≈13 cases have been reported in the literature, and just 1 previous case was described in a patient with HIV co-infection. Our case demonstrates that, even in HIV infection, syphilis manifestations can present as a localized anterior segment mass rather than diffuse ocular inflammation. Internists and infectious disease physicians should be aware that an unexplained iris mass, particularly in patients at risk for sexually transmitted infections, could represent syphilis. Prompt serologic testing and appropriate parenteral therapy are essential to preventing irreversible visual and neurologic complications.

Appendix Additional information on a vascularized iris mass as sentinel manifestation of syphilis in patient with HIV infection, Spain, 2025.
