# Supplementary material for: Vascularized Iris Mass as Sentinel Manifestation of Syphilis in Patient with HIV Infection, Spain, 2025
Source: Emerg Infect Dis. 2026 Jul;32(7):1163–6. doi: 10.3201/eid3207.260388 (PMC13322427; doi:10.3201/eid3207.260388)
Supplement: Appendix — Additional information on a vascularized iris mass as sentinel manifestation of syphilis in patient with HIV infection, Spain, 2025. [file 26-0388-Techapp-s1.pdf]

Article DOI: https://doi.org/10.3201/eid3207.260388

EID cannot ensure accessibility for supplementary materials supplied by authors. Readers who have difficulty accessing supplementary content should contact the authors for assistance.

Vascularized Iris Mass as Sentinel Manifestation of Syphilis in Patient with HIV Infection, Spain, 2025

Appendix

Appendix Table. Summary of published cases of syphilitic vascularized iris masses, 1915–2025*

Case	Author (year)	Age, y/sex	HIV	Lat.	Iris quadrant/ location	AC Cell / KP	CE / PS	Nodule type	UBM	AS-OCT	Initial VA; final VA	Treatment	Resolution
1	Weidler (1915)	45/M	NR	OS	IN / Pupillary margin	NR / NR	CE+ / PS (Poor view)	Papulosa/ Nodosa	NR	NR	CF 3ft; 20/70	Iodid-mercury/ neosalvarsan	75% reduction (1 mo)
2	Weidler (1915)	23/F	NR	OD	ST / Pupillary margin	None / None	CE- / PS+	Papulosa	NR	NR	20/40; NR	Iodid-mercury/ neosalvarsan	Full resolution
3	Weidler (1915)	26/M	NR	OD	IT / Iris base	NR / Hazy aqueous	CE (Hazy) / PS+	Roseola/ Papulosa	NR	NR	NR; NR	Iodid-mercury/ neosalvarsan	Full resolution
4	Schwartz (1980)	30/M	NR	OD	Multiple / Pupillary margin	3+ Cell / KP+	CE- / PS+	Papulosa	NR	NR	20/20; NR	Penicillin/topical steroids	Full resolution
5	McCarron (1984)	34/M	NR	OD	IT / Iris base	2+ Cell / Mutton-fat KP	CE- / PS (NR)	Gummata	B-scan: Ciliary body enlargement	NR	NR; NR	IV Penicillin	Full resolution
6	McCarron (1984)	34/M	NR	OD	IT / Iris base	2+ Cell / NR	CE- / PS-	Gummata	B-scan: Ciliary body involvement	NR	20/25; 20/25	IV Penicillin	Full resolution
7	Tamesis (1990)	34/M	–	OD	T / Iris base	2+ Cell / Mutton-fat KP	CE- / PS (NR)	Gummata	B-scan: Ciliary body involvement	NR	20/25; 20/20	IV Penicillin	Full resolution
8	Yang (2012)	30/M	+	OS	IN / Pupillary margin	Present / NR	CE+ / PS+	Gummata	NR	NR	20/800; 20/400	IV PCN/steroids	Partial resolution
9	de Jong (2017)	40/M	–	OD	IN / Iris base	Present / NR	CE+ / PS-	Gummata	Echodense mass; full-width iris involvement	NR	20/20; NR	IV PCN/steroids	Nearly full resolution

Case	Author (year)	Age, y/sex	HIV	Lat.	Iris quadrant/ location	AC Cell / KP	CE / PS	Nodule type	UBM	AS-OCT	Initial VA; final VA	Treatment	Resolution
10	Michaelov (2018)	53/F	–	OD	ST / Iris base	1+ Cell / Fine KP	CE- / PS-	Gummata	Homogeneous mass protruding from ciliary body	NR	20/50; 20/50	IV Penicillin	Full resolution
11	Chen (2023)	60/M	–	OS	N / Pupillary margin	3–4+ Cell / Mutton-fat	CE+ / PS+	Papulosa	Echodense anterior mass; internal vascular lumens	Hyperreflective surface; vascular lumens; shadowing	20/60; 20/40	IV PCN/steroids	Lost to follow-up
12	Rosenberg (2024)	40/F	–	OS	N / Pupillary margin	2+ Cell / Fine KP	CE- / PS+	Papulosa	Hyperechoic stromal thickening; small lumens	Hyperreflective mass; hyporeflective rim (capsule)	20/70; 20/30	IV/IM PCN/steroids	Full resolution
13	Gonzalez (2025)	73/M	–	OS	Nasal / Pupillary margin	4+ Cell / Mutton-fat	Hypopyon / PS (NR)	Papulosa	NR	SS-OCT: Hyperreflective surface and shadowing	CF; 20/40	IV Penicillin	Full resolution
14	Caminal (2026)	51/M	+	OD	SN / Iris base	3+ Cell / Mutton-fat	CE+ / PS+	Papulosa	Hyperechoic mass; ciliary body involvement; RPE disruption	Hyperreflective mass; RPE disruption; posterior shadowing	HM; 20/20	Ceftriaxone/IV PCN/Steroids	Full resolution

AC, anterior chamber cells; CE, corneal edema; CF, counting fingers; HM, hand motion; IN/IT/ST/SN, inferonasal, inferotemporal, superotemporal, superonasal; KPs, keratic precipitates; NR, not reported; OD, right eye; OS, left eye; PCN, penicillin G; PS, posterior synechiae; VA, visual acuity; +, positive; –, negative.
